# Technique and Muscle Preferences for Dynamic Facial Reanimation in Irreversible Facial Paralysis—A Literature Review

**DOI:** 10.3390/cmtr18010020

**Published:** 2025-03-05

**Authors:** Hilde Schutte, Robbin Maat, Marvick S. M. Muradin, Antoine J. W. P. Rosenberg

**Affiliations:** University Medical Center Utrecht, Heidelberglaan 100, 3584 CX Utrecht, The Netherlands

**Keywords:** facial paralysis, bells palsy, plastic surgery procedures, surgery, plastic, facial muscles, review

## Abstract

Study design: literature review. Introduction: Irreversible facial paralysis is a chronic condition characterized by an absence of mimetic muscle tone and function. This disruption of facial expressions not only has functional, but also psychological and social consequences. In facial dynamic reanimation, techniques are proposed partly recovering facial reanimation and function. To date, a vast amount of literature is available on dynamic reanimation techniques. However, no review has yet been published that delineates in detail the musculature and techniques used for dynamic reanimation in irreversible facial paralysis. Objective: the aim of the present paper is to offer a complete overview of possible techniques. Methods: A chapter division is made between the lower, mid-, and upper face. Each chapter is subdivided between local transposition, free muscle flaps, and for the upper face, implantable devices. Results: The literature discussing reanimation of the lower face is limited. In midfacial reanimation, temporalis transposition and gracilis free flap transfer are popular. In upper facial reanimation, no consensus on muscle choice is available, and information is limited too. Suggested techniques include orbicularis oculi transpositioning, temporalis transpositioning, and platysma free muscle transfer. Conclusions: This paper discusses the current techniques for dynamic facial reanimation. Yet, studies comparing different techniques are lacking, setting ground for future research. This paper highlights the importance of a personalized approach in selecting a fitting reconstruction method.

## 1. Introduction

The facial nerve, also known as the seventh cranial nerve, is of great importance for the functionality of the face. Besides parasympathetic and sensory tasks, the facial nerve contributes predominantly to the innervation of the facial musculature specifically important for facial animation [1]. In facial nerve paralysis, the transmission of signals from the nerve is absent. This can have a central or peripheral cause. Peripheral nerve damage includes traumatic, infectious, tumorous, and idiopathic causes [2]. A 2011 systematic review by Hohman and Hadlock reported that 38% of facial paralysis is idiopathic, often called Bell’s palsy. Bell’s palsy has a lifetime prevalence of 1 in 60 and an annual incidence of 20 per 100,000 persons. The peak incidence is between the ages of 15 and 40. Bell’s palsy is characterized by a sudden onset of unilateral wrinkle loss, ptosis, reduction in the nasolabial fold, and a decrease in the oral commissure. In addition, other facial nerve functions can become aberrant, such as hyperacusis, dry eye and dry mouth formation, and ipsilateral pain dorsal to the ears. In cases of vestibulocochlear nerve infection like Ramsey hunt syndrome, facial paralysis is also accompanied by tinnitus, vertigo, and loss of hearing. Although the majority of Bell’s palsy is reversible, 13 percent sustains mild paresis, and 4 to 5 percent show noticeable prolonged facial dysfunction [3,4,5].

Facial paralysis is associated with a negative and dismal appearance. Additionally, patients are perceived as less intelligent, trustworthy, and attractive. Furthermore, their own perception is affected, reporting more depression, a lower quality of life, and worse mood than the control group [6,7]. This stresses the importance of the preservation of facial mimics with dynamic surgical methods. Several surgical reanimation options are available for irreversible facial paralysis, depending on the extent of the disease. If the muscles and motor endplates are still viable, paralysis might be corrected by means of neurotization (nerve grafts, nerve transfers, etc.). Irreversible degeneration of the muscles calls for the use of muscle transposition, muscle transfers, or a combination of muscle grafts and nerve grafts or nerve transfers [8,9]. Facial reanimation can be categorized into three geographical facial regions: firstly, the upper lip and midface; secondly, the lower lip; and thirdly, the ocular region. Currently, a vast amount of literature is available on dynamic reanimation techniques. However, no review has yet been published that overviews all the musculature and techniques used for dynamic reanimation in irreversible facial paralysis. Therefore, the aim of this paper is to give a detailed overview of the muscles and techniques used for dynamic facial reanimation in irreversible facial paralysis.

To give a comprehensive overview, a chapter division is made between lower-, mid-, and upper-face reanimation techniques based on anatomical regions (Table 1). Each chapter subsequently describes the available techniques for dynamic reanimation in that specific facial region, distinguishing between local transpositions and muscle transfers. For upper face reanimation, a side step is made towards active implantable prostheses. In local transpositions, a muscle within the primary surgical site is (partly) dissected and rotated or repositioned to fulfill the function of the paralyzed muscle. In free muscle transfer techniques, the muscle is harvested from its anatomical site, with the inclusion of its vasculature and nerve branches, and is then reinserted, revascularized, and reinnervated on the face.

## 2. Lower Face Dynamic Reanimation Techniques

When smiling, besides oral commissure elevation, lower lip depression occurs. Under normal circumstances, the depressor angularis oris, the depressor labii inferioris, and the mentalis muscle contribute to lower lip depression. Consequently, in unilateral facial paralysis, asymmetry during smiling occurs. Lower lip reanimation surgery aims to restore this deficit.

### 2.1. Local Transposition Techniques

#### 2.1.1. Platysma Muscle Transposition

In some cases of facial paralysis, for example, in Bell’s palsy, the cervical branch of the facial nerve is not affected. This branch contributes to the innervation of the platysma [2,10]. On these occasions, the platysma is of first choice for depressor reanimation. During surgery, the muscle is partly dissected, followed by a flap rotation in order to eventually be attached to the inferior margin of the orbicularis oris [10,11,12].

#### 2.1.2. Digastric Muscle Transposition

After harvesting the complete muscle, the proximal end of the graft is split, enabling a spread-out tethering to the lower margin of the orbicularis oris muscle [13,14].

### 2.2. Free Muscle Transfer Techniques

#### Gracilis-Combined Muscle Flap

In this procedure, a gracilis flap is harvested, followed by a partial longitudinal section, creating two flaps. The larger muscle flap is used for midface reanimation, and the smaller muscle flap is used for lower lip reanimation [15].

## 3. Discussion Lower Face

The extent of facial paralysis determines which muscle could be used. If the cervical branch of the facial nerve is not affected and the platysma is still functioning, this muscle is preferred for depressor reanimation. However, if the platysma is affected, digastric muscle transposition is the next viable option [2,10,11,12,13,14,15,16,17].

## 4. Midfacial Dynamic Reanimation Techniques

Normally, the zygomatic muscle predominantly causes retraction and elevation of the corners of the mouth, resulting in the typical smile [18]. Restoring that function is the main goal of midface reanimation.

### 4.1. Local Transposition Techniques

#### 4.1.1. Masseter Muscle Transposition

Usually, the masseter muscle is partly released from its insertion on the mandible and is reinserted to the orbicularis oris muscle. The advantages of the masseter muscle are its size, accessibility, and proximity to the lips. An additional positive aspect of using this muscle for reanimation surgery is that the masseter is already thought to play a physiological role in smile animation [19]. As the pterygoid muscle can cover the masseter function, donor site morbidity is minimal [20]. A downside of the technique is the resultative horizontal vector of the transposed muscle fibers. This hampers an effective alleviation of the oral commissure. Other disadvantages described are a short muscle reach, a postoperative mandibular concave deformity, the possibility of trismus or difficulties with mastication, and postoperative facial asymmetry. Therefore, adaptations have been developed. With a pedicled masseter muscle transposition, the masseter muscle can be both released from its origin and insertion and can be routed in the direction of the zygomaticus muscle. This results in a more accurate muscle vector for smile animation. Moreover, some papers report the use of a fascia lata sling, which is ligated to the masseter muscle, passed over the zygomatic arch, and then ligated to the orbicularis oris muscle. In this way, the zygomatic arch functions as a pulley, elevating the oral commissure when the masseter muscle is flexed [20,21,22].

#### 4.1.2. Temporalis Muscle Transposition

Physiologically, the temporalis muscle functions as an elevator and protrudor of the mandible. After transposition, these functions can be covered by the pterygoid muscle [20,23]. Contemporary surgical techniques for temporalis muscle transposition include three options: temporalis tendon transpositioning with temporalis lengthening myoplasty (the so-called SMILE technique), temporalis tendon transpositioning without lengthening myoplasty, and the mini flap procedure.

○ Temporalis tendon transpositioning with temporalis lengthening myoplasty (SMILE technique)

In temporalis lengthening myoplasty, the temporalis muscle is released from its posterior and inferior origin at the cranium, thereby reducing muscle tension. Next, the temporalis is released from its coronoid insertion and relocated to the oral commissure. Thereafter, the origins are restored concomitantly, restoring muscle tension. Depending on the procedure, the zygomatic arch could be segmented [24].

○ Temporalis tendon transpositioning without temporalis lengthening myoplasty

In this technique, cranial origin sites are only partly dissected. In order to achieve length, fascia lata grafts are used. Fascia lata grafts can be tunneled to the upper and lower lip. For a more widespread insertion, the nasolabial fold can also be used as a tethering point [25,26].

○ Mini Flap Procedure

This technique preserves temporalis muscle physiology by creating a mini flap just dorsal to the coronoid process by means of a U-shaped incision. The lengthy gap is bridged by a fascia lata graft [27].

### 4.2. Free Muscle Transfer Techniques

For facial reanimation, different muscle grafts have been proposed. Factors to determine the suitability of a muscle include donor site morbidity, vascular stability, muscle length, and the type and form of the muscle fibers [28,29].

#### 4.2.1. Gracilis Free Muscle Transfer

In 1976, Harii et al. initially proposed the gracilis free muscle flap for midface reanimation [30]. The advantages of this technique are that it is a reliable technique since there are only a few anatomical variants, that the identification of the vasculature is easy, with appropriate options for anastomosis, and that the muscle fiber pattern is parallel instead of pennate, causing a longer excursion length. Moreover, donor site morbidity is low or even absent due to the fact that scarring is easy to conceal, and the muscle’s function is versatile. Reported disadvantages of this technique are the bulkiness of the muscle, which can result in unaesthetic deformity, the fact that that it has slow twitch instead of fast twitch muscle fibers, and that the proximal tendon is short, making muscle tethering more complex [28,29]. Several muscles have been proposed as alternatives for the gracilis. Yet, the gracilis free muscle flap remains the gold standard ever since Harii initially described this technique in 1976 [31,32].

#### 4.2.2. Latissimus Dorsi Free Muscle Transfer

The latissimus dorsi, just like the gracilis, has parallel muscle fibers, which are associated with a large contractile length. The gracilis muscle in situ is superior in contractility over the latissimus dorsi. However, after CFNG, no significant difference between the excursion of the gracilis and the latissimus dorsi was seen [33]. The latissimus does excel in a shorter recovery period and the one-stage surgery in comparison to the gracilis muscle transfer. Additionally, donor site morbidity is low [34].

#### 4.2.3. Serratus Anterior Free Muscle Transfer

The serratus anterior muscle contains a reliable neurovascular pedicle, which makes it possible to graft five muscle flaps out of one neurovascular pedicle. This enables the muscle to function as a multivector graft implant, ideal for facial animation. Both CFNG and ipsilateral neurotization is possible. A small disadvantage might be the fan shape of the muscle, preventing a centered oral commissure pull. Additionally, compared to the gracilis, the force vector is low [35,36].

#### 4.2.4. Sternohyoid Free Muscle Transfer

An advantage of using the sternohyoid muscle is that its surgical area is familiar territory for the maxillofacial surgeon. Furthermore, vascularization and innervation are easy to access and have sufficient lengths, making the use of intermediate nerve grafts unnecessary [37]. Additionally, the fast twitch fiber ratio of the sternohyoid muscle is comparable to the zygomaticus, resulting in a favorable length–contraction ratio [38]. Finally, the muscle is less bulky, which has aesthetic advantages [39]. Disadvantages include that first, the hyoid bone is included in the graft, resulting in increased morbidity and increased complexity of graft anchoring to the zygomatic bone. More specifically, osteosynthesis material is needed, which might induce a zygomatic bulk formation. Also, muscle shortening is difficult because of the vascular bundle and the nerve fibers that are situated on opposite ends [28,37,39].

#### 4.2.5. Pectoralis Minor Free Muscle Transfer

The pectoralis minor muscle is favorable because of the fan-shaped contours, good tendinous anchor points, and small muscle scale, with low donor site morbidity [40].

#### 4.2.6. Coracobrachialis Free Muscle Transfer

A lot of features of the coracobrachialis muscle are comparable with the gracilis muscle. For example, both have reliable vascular supply, which can be used for anastomosis. Additionally, both muscles have little to no donor site morbidity nor suffer from functional impairment after harvesting. However, the coracobrachialis exceeds in its slim form, demanding near to no debulking. Moreover, the shape allows it to be firmly anchored to its origin and insertion. However, to the best of our knowledge, the only literature concerning this technique is dated 2003 by Taylor et al. [29].

#### 4.2.7. Abductor Hallucis Longus Free Muscle Transfer

The advantages of the hallucis longus muscle are that the muscle functions parallel to normal facial musculature, no remarkable functional impairment of the donor site is encountered, and reliable neurovascular patterns are provided. Moreover, graft collection and facial preparation can be performed simultaneously, as is the case in the gracilis and serratus anterior muscle flaps [41].

## 5. Discussion: Midfacial Reanimation

The gold standard for midfacial reanimation is the gracilis free muscle transfer [30,31]. Yet, many other options for free muscle transfers have been proposed, including the latissimus dorsi, the serratus anterior, the sternohyoid, the pectoralis minor, the coracobrachialis flap, and the abductor hallucis longus muscle flap [28,29,33,34,35,40,41]. Factors to determine the suitability of a muscle include donor site morbidity, vascular stability, muscle length, and the type and form of the muscle fibers [28,29]. Local transposition techniques have been described as well, using the masticatory muscles, with a preference for the temporalis over the masseter [19,20,42].

Local reanimation techniques make use of muscles that are not innervated by the facial nerve. Because of that, spontaneous smiling, as a postoperative result, requires extensive smile rehabilitation [43]. In free tissue transfer, however, spontaneous smiling can be achieved with the use of CFNG, most of the time connecting an intermediate sural nerve graft that is connected to the muscle graft to branch off to the non-paralyzed contralateral zygomatic or buccal nerve in the process partly sacrificing these nerves. A noticeable side-effect, therefore, could be partial postoperative nerve function impairment on the non-paralyzed facial side [44,45].

For the choice of the right technique, one has to take into consideration the aim of the operation. Where temporalis tendon transposition is known to profoundly restore resting symmetry, gracilis muscle transfer is known to achieve more reliable facial animation and allow for spontaneous smiling. Also, the difference in recovery time is longer in gracilis muscle transfer surgery, whereas the temporalis transposition takes 0–3 months to see oral commissure excursion improvements, and gracilis transfer takes at least 3–6 months, even prolonging to 12 months. Decision-making is dependent on the paralysis etiology, the life expectancy, the need for fast recovery, and the preoperative state of the paralysis [23]. The age of the patient is an important factor, too. Younger patients, having higher regenerative potential, are more suitable for gracilis free flaps than the elderly, where static procedures would show the same static results and have less morbidity.

## 6. Upper Face Dynamic Reanimation Techniques

One way of classifying smiling is to distinguish between a Duchenne smile and a non-Duchenne smile, also known as a genuine smile or a non-genuine smile. A Duchenne smile is a coordinated interplay between contraction of the zygomaticus major and orbicularis oculi muscles, resulting in a raised oral commissure and an increased nasolabial fold in combination with eye wrinkling. Thus, to restore genuine smiling in facial paralysis, restoration of periocular dynamics is required [18]. Besides aiding in smile animation, the orbicularis oculi muscle enables blinking and eyelid closure. This is essential for corneal protection and tear drainage; shortcomings cause irritation of the eye, reflex tearing, keratitis, keratopathy, conjunctival edema, and, in the worst case, blindness [46]. In irreversible facial paralysis, dynamic reanimation techniques focus on restoring eyelid closure and blinking, as well as on restoring a genuine smile. Again, local transposition and free muscle flaps will be discussed, but this chapter also includes the use of active implantable devices.

### 6.1. Local Transposition Techniques

#### 6.1.1. Temporalis Transposition

During this procedure, a centrally located temporalis flap is created. To the flap, a split fascia lata sling is attached. The inferior sling is placed quite superficially, directly under the upper margins of the lower eyelid, so that the ectropion is erased. The superior sling is placed in a bow-like manner to maximize upper eyelid closure and minimize a split-like appearance due to too much tension. Zygomatic bulging is prevented by soft tissue removal [47]. The advantages of the procedure are the low complexity, use of a local muscle, one-stage design, and fast recovery of function. The disadvantages of the procedure are the resultative lateral instead of vertical pull on the eyelids, incapacity of restoring automatic blinking, eyelid movement when chewing, secondary mechanical ptosis, bulk formation on the eyelids, nocturnal lagophthalmos, and sometimes ectopic bone formation or temporomandibular joint dysfunction [23,46,47,48,49].

#### 6.1.2. Orbicularis Oculi Transposition

A relatively novel technique is the contralateral orbicularis oculi muscle transposition. In this technique, a rectangular flap is created, leaving only the nasal part of the flap attached to the orbicularis oculi muscle. This flap is literally split, followed by a subcutaneous tunneling of the muscle flap to the contralateral side. After rotation of the orbicularis muscle flap, the two lids are fixed to the upper and lower eyelids. An advantage of orbicularis oculi transposition is the innervation by the contralateral facial nerve. This results in a natural, coordinated, and fast postoperative regeneration of blinking, and nocturnal closed eyelids. Donor site morbidity appears to be minimal [50,51].

#### 6.1.3. Frontalis Transposition

For frontalis transposition, the contralateral muscle is tunneled while keeping its original vascularisation and innervation [47]. It is also possible to use the ipsilateral muscle with a two-step procedure. First, a CFNG is connected from an intact facial nerve branch towards the contralateral paralyzed side, in some cases temporarily connecting the CFNG to the contralateral hypoglossal nerve. Second, one year later, the ipsilateral frontalis muscle is partly transferred toward the upper and lower eyelids to substitute for the orbicularis oculi muscle [52,53].

### 6.2. Free Muscle Transfer Techniques

#### 6.2.1. Platysma Free Muscle Transfer

The advantages of the platysma are the thinness, the consistent nerve entry location, and the consistent submental artery supply [54,55,56]. Possible disadvantages are the difference regarding biomechanical properties compared to the orbicularis oculi and oral commissure dropping due to arterial dissection at the level of the labial artery bifurcation in order to create sufficient pedicle length. Furthermore, it is important to note that better results are obtained when surgery is performed before the age of 30 [55].

#### 6.2.2. Gracilis-Combined Free Muscle Transfer

The technique used in gracilis muscle transfer addresses both the midface and upper face reanimation. In this technique, a composite gracilis graft provides one arm for ocular contraction, and one arm is used for oral commissure elevation [57,58].

#### 6.2.3. Occipitalis Free Muscle Transfer

The advantages of this technique are that the occipitalis has favorable conditions for transfer, including its excursion, tension, thickness, and innervation density. Yet, the harvest is challenging because of the complexity of the region and the variable dimensions of the posterior auricular nerve, and transfer requires preparation in the anatomy laboratory [52].

#### 6.2.4. Extensor Digitorum Brevis Free Muscle Transfer

The extensor brevis has been described for blink restoration in the early 1970s. Yet, no reproduction of these results has been reported ever since [59].

### 6.3. Active Implantable Prostheses

#### 6.3.1. Direct Electrical Stimulation

This technique uses implantable electrodes that stimulate the paralyzed muscle. Therefore, a functioning neuromuscular unit is required [60,61].

#### 6.3.2. Electro-Active Polymers

Electro-active polymers function as artificial muscle. The implanted polymer has dielectric properties, and when it is placed between two polarized electrodes, it contracts [62]. This technique seems promising in restorating facial paralysis in animals, yet it still has to prove its applicability in humans [63].

## 7. Discussion: Upper Face Reanimation

The gold standard for adults is the local transposition of the temporalis [23,64]. For children, the gold standard is the gracilis-combined free muscle transfer, as temporalis transposition is thought to cause skeletal growth impairment when performed at a young age [23]. The temporalis transposition and frontalis transposition show (equally) minor improvements in children [53].

## 8. General Conclusions

This paper provides a broad overview of the current techniques used in dynamic facial reanimation, focusing on their indications, advantages, and limitations. Despite the increasing amount of literature on facial reanimation, there remains a notable lack of high-quality comparative studies evaluating the efficacy of different techniques. The absence of direct comparisons makes it challenging to establish clear guidelines for optimal surgical approaches, underscoring the need for future research in this field.

Furthermore, there is significant variability in the presentation of facial palsy among patients, with differences in etiology, severity, duration, and individual factors playing a crucial role in determining the most effective treatment. As a result, a standardized, one-size-fits-all approach is not suitable for dynamic facial reanimation. Instead, this paper highlights the importance of a patient-specific strategy, in which the choice of reconstruction method is tailored to the unique clinical presentation and functional goals of each individual. By advocating for a more personalized approach, we aim to encourage further investigation into patient-specific outcomes and the development of refined selection criteria for different reanimation techniques.

## Figures and Tables

**Table 1:** Muscle preferences in irreversible facial paralysis. A Schematic portrayal of techniques used for dynamic facial reanimation. The face is segmented into the upper, mid, and lower face. Also, a subdivision is made between local transpositioning and free muscle transferring techniques.

1: Lower face reanimation	a. Local transpositioning Platysma muscle transpositioning Anterior belly of the digastric muscle transpositioning
	b. Free muscle transfer Combined lower and midface gracilis muscle transfer
2: Midface reanimation	a. Local transpositioning Masseter muscle transpositioning Temporalis muscle transpositioning: - Temporalis lengthening myoplasty - Temporalis non-lengthening myoplasty - Mini flap procedure
	b. Free muscle transfer Gracilis muscle transfer Latissimus dorsi Serratus anterior Sternohyoid Pectoralis minor Coracobrachialis Abductor hallucis longus
3: Upper face reanimation	a. Local transpositioning Temporalis muscle and fascia transpositioning Contralateral orbicularis oculi transpositioning Frontalis transpositioning
	b. Free muscle transfer Platysma muscle transfer Gracilis muscle transfer Occipitalis free muscle transfer Extensor digitorum brevis free muscle transfer
	c. Active implantable prostheses Direct electrical stimulation Electro-active polymers

## Data Availability

Not applicable.
